# Off‐target effects of bacillus Calmette–Guérin vaccination on immune responses to SARS‐CoV‐2: implications for protection against severe COVID‐19

**DOI:** 10.1002/cti2.1387

**Published:** 2022-04-22

**Authors:** Nicole L Messina, Susie Germano, Rebecca McElroy, Rajeev Rudraraju, Rhian Bonnici, Laure F Pittet, Melanie R Neeland, Suellen Nicholson, Kanta Subbarao, Nigel Curtis, the BRACE trial, Adrian Siles Baena, Jia Wei Teo, Guilherme Teodoro de Lima, Carolinne Abreu, Veronica Abruzzo, Lynne Addlem, Sophie Agius, Adelita Agripina Refosco Barbosa, Ahmed Alamrousi, Ayla Alcoforado da Silva dos Santos, Yasmeen Al‐Hindawi, Samyra Almeida Da Silveira, Lais Alves da Cruz, Jeremy Anderson, Amenyogbe Nelly, Christina Anthony, Andrea Antonia Souza de Almeida dos Reis Pereira, Francisco Arnaiz de las Revillas Almajano, Annabelle Arnold, Beth Arrowsmith, Kristy Azzopardi, Cristina Badia Marti, Twinkle Bahaduri, Samantha Bannister, Sarah Barney, Lydia Barrera, Anabel Barriocanal, Dayanne Barros, Simone Barry, Adam Bartlett, Lilian Batista, Silva Muranaka, Therese Baulman, Morgan Bealing, Justin Beardsley, Ana Belen Martin Gutierrez, Jason Bell, Saoirse Benson, Benothman Rym, Vicki Bennett‐Wood, Nikki Bergant, Fabiane Bianca Barbosa, Wouter Bijllaardt, Patricia Bimboese, Camila Bitencourt de Andrade, Stephen Blake, Kitty Blauwendraat, Wim Boersma, Pilar Bohedo Garcia, Marc Bonten, Anne Boon, Anna Bourke, Kirsty Bowes, Larissa Brasil, Clare Brophy, Rochelle Botten, Sandy Buchanan, Jess Bucholc, Alison Burns, Emma Burrell, Natalia Bustos, Bridie Byrne, Anthony Byrne, Esther Calbo, Jorge Calvo Montes, Beatriz Camesella, John Campbell, Atsegiñe Cangas, John Carlin, Maria Carmen Roque, Roberta Carolina Pereira Diogo, Estela Carvalho, Irma Casas, Erika Castro, Ramon Castro, Helen Catterick, Rodrigo Cezar Dutra Escobar, Joyce Chan, Jo Cheah, Tee Yee Chern, Thilakavathi Chengodu, Marianna Ciaverella, Sharon Clark, Marie‐Alix Clement Espindola, Annie Cobbledick, Clinton Colaco, Simone Collopy, Patricia Comella, Mary Corbett, Gabriela Correa E Castro, Erlane Costa, Raquel Coya, Nigel Crawford, Julio Croda, Alda Cruz, Curtis Maxwell, Jac Cushnahan, Anna Czajko, Renato da Costa Silva, Bouchra Daitiri, Margareth Dalcolmo, Karen Dalton, Thanh Dang, Aiken Dao, Andrew Davidson, Phoebe Dawe, Diane Dawson, Miriam de Jesus Costa, Karina De La Cruz, Almudena de la Serna, Fabiani de Morais Batista, Adriely de Oliveira, Rocio del Alba Rey Morillo, Maria Desylva, Helga Dijkstra, Rachel Dixon, Maria Dolores del Toro Lopez, Jose Dominguez, Catriona Doran, Angel Dominguez Castellano, Glauce Dos Santos, Joyce dos Santos Lencina, Débora dos Santos Silva, Mark Douglas, Ross Dunn, Andrew Dunn, Jemma Dunnill, Georgina Eden, Edler Peta, Harriet Edmund, Nat Eiffler, Hannah Elborough, Sonja Elia, Olivia Elkington, Michelle England, Wellyngthon Espindola Ayala, Maria Esteve, Nick Evans, Sue Evans, Krist Ewe, María Carmen Fariñ Álvarez, Kieran Fahey, Jill Fairweather, Denise Faustman, Erica Fernandes Silva, Monique Fernandez, Galina Fidler, PMG Filius, Adam Finn, Carolyn Finucane, Stephanie Firth, Emily Fletcher, Catherine Flynn, Lorraine Flynn, Liam Fouracre, Sarah Fowler, Thamires Freitas, Ana Carolina Furtado, Maria Gabriela Oliveira, Anna Gabriela Santos, Leandro Galdino Cavalcanti Gonçalves, Laura Galletta, Larissa Gama, Dinusha Gamage, Radhika Ganpat, Carlos García, Mariana Garcia Croda, Kaya Gardiner, Evangeline Gardiner, Grace Gell, Aline Gerhardt de Oliveira, Susie Germano, Michael Gibbons, Camille Gibson, Alison Gifford, Teresa Giménez Poderos, Ann Ginsberg, Jet Gisolf, Bojana Gladanac, Penny Glenn, Vanessa Godinho, Mayara Góes dos Santos, Josune Goikoetxea, Telma Goldenberg, Adriano Gomes, Susana Gonzalez Marcos, Claudia González Rico, Casey Goodall, Louise Goodchild, Victoria Gordon, Frances Greven, Ana Greyce Capella, Liddy Griffith, Christina Guo, David Gutierrez Campos, Manuel Gutierrez Cuadra, Amanda Gwee, Richard Hall, Lydia Hall, Kate Hamilton, Matthew Hannan, Houda Harbech, Alex Harding, Neil Harker, Robert Harrison, Robert Jan Hassing, Thaynara Haynara Souza da Rosa, Zaheerah Haywood, Christine Heath, Nadine Henare, Paulo Henrique Andrade, Susan Herrmann, Erin Hill, Sam Hilton, Danique Huijbens, Heidi Hutton, Jane James, Tenaya Jamieson, Axel Janssen, Bruno Jardim, Tyane Jardim, Lance Jarvis, Narelle Jenkins, Jane Jones, Jan Jones, Karen Jones, Leticia Jorge, Maria Jose Rios Vilegas, Sri Joshi, Rosemary Joyce, Joel Junior, Rama Kandasamy, Anushka Karunanayake, Hana Karuppasamy, Tom Keeble, Jennifer Kent, Paul Kloeg, Jan Kluytmans, Bridget Knight, Tobias Kollmann, Tony Korman, Ann Krastev, Meredith Krieg, Nathan La, Marcus Lacerda, Alicia Lacoma, Renier Lagunday, Debbie Lalich, Erin Latkovic, Irene Latorre, Paulo Leandro Garcia Meireles, Katherine Lee, Donna Legge, Toos Lemmers, Titia Leurink, Katherine Lieschke, Kee Lim, Gemma Lockhart, Cíntia Lopes Bogéa, Karla Lopes dos Santos, Reyes Lopez Marques, Michaela Lucas, David Lynn, Miriam Lynn, Maria Luciana Silva De Freitas, Norine Ma, Sam Macalister, Cristiane Machado, Matheus Machado Ramos, Francesca Machingaifa, Ivan Maia, Bernardo Maia, Richard Malley, Laurens Manning, Sarah Manton, Jose Manuel Carrerero, Ana Maria Barriocanal, Cíntia Maria Lopes Alves, Rosa Maria Plácido Pereira, Bianca Maria Silva Menezes Arruda, Adriana Marins, Angela Markow, Helen Marshall, Christopher Martin, Katya Martinez Almeida, Wayne Mather, Megan Mathers, Fábio Mauricio Nogueira Gomes, Mariana Mayumi Tadokoro, Nadia Mazarakis, Kelry Mazurega, Sonia McAlister, Amy McAndrews, Ellie McDonald, Fiona McDonald, Mark McMillan, Brendan McMullan, Nick McPhate, Lee Mead, Andrea Meehan, Bob Meek, Rosangela Melo, Guillermo Mena, Daniella Mesquita, Isabella Mezzetti, Hugo Miguel Ramos Vieira, Skye Miller, Kirsten Mitchell, Marcus Mitchell, Jesutofunmi Mojeed, Kitty Molenaar, Gemma Molina, Barbara Molina, Lisa Montgomery, Cecilia Moore, James Moore, Simone Moorlag, Thilanka Morawakage, Julie Moss, Will Moyle, Kim Mulholland, Craig Munns, Elizandra Nascimento, Nicolas Navarrette, Mihai Netea, Juliana Neves, Georgina Newman, Belle Ngien, Jill Nguyen, Khanh Nguyen, Fran Noonan, Wendy Norton, Melissa O’Donnell, Jess O’Bryan, Abby O’Connell, Sasha Odoi, Liz O’Donnell, Roberto Oliveira, Marilena Oliveira, Thais Oliveira, Ingrid Oliveira, Nadia Olivier, Ligia Olivio, Benjamin Ong, Jaslyn Ong, Joanne Ong, Jakob Onysk, Isabelle Ooi, Frances Oppedisano, Francesca Orsini, Belinda Ortika, Arthur Otsuka, Kristen Overton, Rosie Owens, Rayssa Paes, Pamela Palasanthiran, Virginia Palomo Jiménez, Girlene Pandine, Kimberley Parkin, Alvaro Pascual Hernandez, Nienke Paternotte, David Paterson, Ana Paula Conceição de Souza, Lisa Pelayo, Casey Pell, Sille Pelser, Handerson Pereira, Gabrielle Pereira, Glady Perez, Cristina Perez, Tomás Perez Porcuna, Susan Perlen, Kirsten Perrett, Amandine Philippart De Floy, Sigrid Pitkin, RC Pon, Ines Portillo Calderón, Jeffrey Post, Catherine Power, Christiane Prado, Endriaen Prajitno, Cristina Prat‐Aymerich, Lieke Preijers, Marco Puga, Evelyn Queiroz, Lynne Quinn, Ashleigh Rak, Leticia Ramires Figueiredo, Encarnacion Ramirez de Arellano, Pedro Ramos, Karla Regina Warszawski de Oliveira, Jack Ren, Stephanie Reynolds, Shelley Rhodes, Claudinalva Ribeiro dos Santos, Chris Richards, Peter Richmond, Holly Richmond, Ana Rita Lopes Souza, Jorge Rocha, Teresa Rodrigues, Laleyska Rodrigues, Bebeto Rodrigues, Iara Rodrigues Fernandes, Jesús Rodríguez‐Baño, Nienke Roescher, Sally Rogers, Anke Rol, Jannie Romme, Antoni Rosell, Maria Roser Font, Domenic Sacca, Sonia Sallent, Vanderson Sampaio, Nuria Sanchez, Blanca Sanchez, Daniel Santos, Tilza Santos, Ariandra Sartim, Amber Sastry, Alice Sawka, Nikki Schultz, Clare Seamark, David Seamark, Engelien Septer‐Bijleveld, Raquel Serrano, Frank Shann, Ketaki Sharma, Margaret Shave, Lisa Shen, Kate Sidaway‐Lee, Rafaela Silva, Juliana Silva, Emanuelle Silva, Mariana Simão, Ronita Singh, Marilda Siqueira, Marciléia Soares D. Allão Chaves, Thijs Sondag, Enoshini Sooriyarachchi, Antonny Sousa, Spotswood Judith, Leena Spry, Sarah Statton, Andrew Steer, Dyenyffer Stéffany Leopoldina dos Santos, Katrina Sterling, Leah Steve, Luke Stevens, Natalie Stevens, Carolyn Stewart, Lisa Stiglmayer, Lida Stooper, Josephine Studham, Eva Sudbury, Astrid Suiker, Lorrie Symons, Esther Taks, Niki Tan, Bruna Tayara Leopoldina Meireles, Menno te Riele, Jaap ten Oever, Rob ter Heine, Jhenyfer Thalyta Campos Angelo, Helen Thomson, Ryan Toh, Alexandre Trindade, Harry Tripp, Enriqueta Tristán, Darren Troeman, Alexandra Truelove, Daniel Tsuha, Marlot Uffing, Fernando Val, Olga Valero, Ester Valls, Chantal van de Ven, Leo van Den Heuvel, Sigrid van der Veen, Marije van der Waal, JH Van Leusen, Linda van Mook, H van Onzenoort, Marjoleine van Opdorp, Miranda van Rijen, Nicolette van Sluis, Adria Vasconcelos, Noelia Vega, Sunitha Velagapudi, Louise Vennells, Tamsin Venton, Harald Verheij, P.M. Verhoeven, Caroliny Veron Ramos, Paulo Victor Rocha da Silva, Sandra Vidal, Suzanna Vidmar, Patricia Vieira, Matheus Vieira de Oliveira, Rosario Vigo Ortega, Paola Villanueva, Raquel Villar, Amanda Vlahos, Ushma Wadia, Mary Walker, Kate Wall, Rachael Wallace, Xiaofang Wang, Justin Waring, Ruth Warren, Adilia Warris, Emma Watts, Michelle Wearing‐Smith, Daniel Webber‐Rookes, Jamie Wedderburn, Ashleigh Wee‐Hee, Steve Wesselingh, Bethany Whale, Phoebe Williams, Beatrijs Wolters, Nick Wood, Ivy Xie, Angela Younes, Angela Young, Felipe Zampieri, Vieira Batista, Carmen Zhou, Vivian Zwart

**Affiliations:** ^1^ Infectious Diseases Group, Infection and Immunity Theme Murdoch Children’s Research Institute Parkville VIC Australia; ^2^ Department of Paediatrics The University of Melbourne Parkville VIC Australia; ^3^ Department of Microbiology and Immunology University of Melbourne at The Peter Doherty Institute for Infection and Immunity Parkville VIC Australia; ^4^ 27230 Paediatric Infectious Diseases Unit Faculty of Medicine Geneva University Hospitals Geneva Switzerland; ^5^ Molecular Immunity Group, Infection and Immunity Theme Murdoch Children’s Research Institute Parkville VIC Australia; ^6^ Victorian Infectious Diseases Reference Laboratory The Royal Melbourne Hospital The Peter Doherty Institute for Infection and Immunity Parkville VIC Australia; ^7^ WHO Collaborating Centre for Reference and Research on Influenza Peter Doherty Institute for Infection and Immunity Parkville VIC Australia; ^8^ Infectious Diseases The Royal Children's Hospital Melbourne Parkville VIC Australia

**Keywords:** COVID‐19, BCG, cytokine, T cell, SARS‐CoV‐2, cytokine, immunoregulation

## Abstract

**Background and objectives:**

Because of its beneficial off‐target effects against non‐mycobacterial infectious diseases, bacillus Calmette–Guérin (BCG) vaccination might be an accessible early intervention to boost protection against novel pathogens. Multiple epidemiological studies and randomised controlled trials (RCTs) are investigating the protective effect of BCG against coronavirus disease 2019 (COVID‐19). Using samples from participants in a placebo‐controlled RCT aiming to determine whether BCG vaccination reduces the incidence and severity of COVID‐19, we investigated the immunomodulatory effects of BCG on *in vitro* immune responses to SARS‐CoV‐2.

**Methods:**

This study used peripheral blood taken from participants in the multicentre RCT and BCG vaccination to reduce the impact of COVID‐19 on healthcare workers (BRACE trial). The whole blood taken from BRACE trial participants was stimulated with γ‐irradiated SARS‐CoV‐2‐infected or mock‐infected Vero cell supernatant. Cytokine responses were measured by multiplex cytokine analysis, and single‐cell immunophenotyping was made by flow cytometry.

**Results:**

BCG vaccination, but not placebo vaccination, reduced SARS‐CoV‐2‐induced secretion of cytokines known to be associated with severe COVID‐19, including IL‐6, TNF‐α and IL‐10. In addition, BCG vaccination promoted an effector memory phenotype in both CD4^+^ and CD8^+^ T cells, and an activation of eosinophils in response to SARS‐CoV‐2.

**Conclusions:**

The immunomodulatory signature of BCG’s off‐target effects on SARS‐CoV‐2 is consistent with a protective immune response against severe COVID‐19.

## Graphical abstract

Using samples from participants in a multisite placebo‐controlled RCT, we found that bacillus Calmette–Guérin (BCG) vaccination alters immune responses to SARS‐CoV‐2. The immunomodulatory signature of BCG on SARS‐CoV‐2 responses is consistent with a protective immune response against severe COVID‐19.

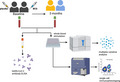

## Introduction

The paradigm of vaccination is the induction of antigen‐specific memory in adaptive immune cells. However, it is increasingly recognised that vaccines can have beneficial off‐target (aka non‐specific or heterologous) effects. One such vaccine is the live‐attenuated tuberculosis (TB) vaccine, bacillus Calmette–Guérin (BCG). Randomised controlled trials (RCTs) show that BCG vaccination protects against respiratory tract infections, with observed reductions of up to 80%.^1^, ^2^, ^3^ The beneficial off‐target effects of BCG are attributed to BCG‐mediated immunomodulation.

**Figure 1:**
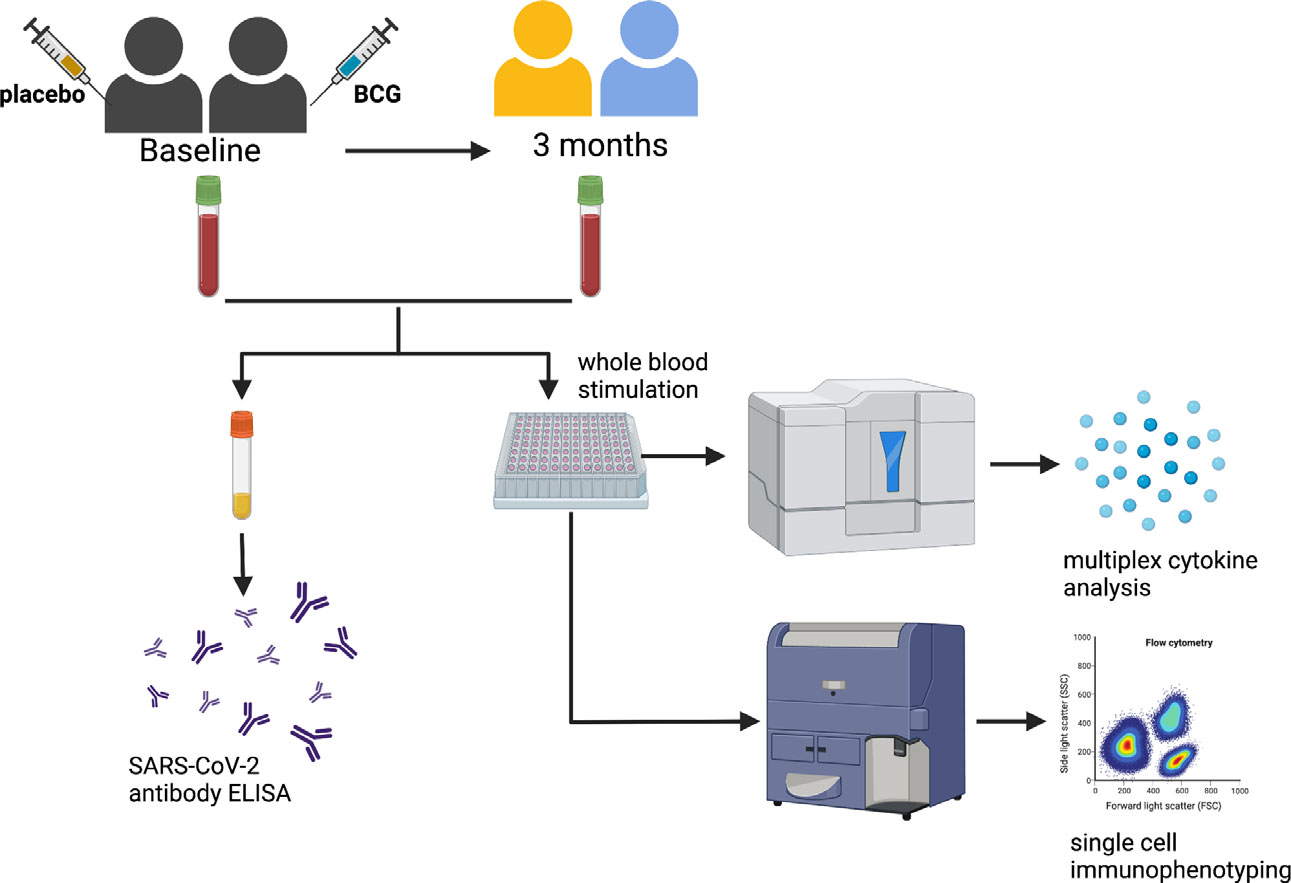
Schematic of study design. Created with BioRender.com.

Given the strong evidence for BCG‐mediated protection against unrelated infections, over 20 clinical trials are underway to determine whether BCG vaccination can protect against COVID‐19.^4^ These studies are based on the premise that BCG vaccination enhances immune responses to SARS‐CoV‐2 to protect against COVID‐19. Although effective COVID‐19‐specific vaccines are now available, because of shortages in supply and the emergence of potential immune‐escape variants, BCG vaccination may offer an easily accessible preventative intervention to bolster protection against infections with SARS‐CoV‐2 or other future novel pathogens until specific vaccinations can be given. Using samples from SARS‐CoV‐2‐naïve participants in a multicentre RCT and BCG vaccination to reduce the impact of COVID‐19 in healthcare workers (BRACE trial; NCT04327206),^5^ we investigated whether BCG vaccination alters *in vitro* immune responses to SARS‐CoV‐2 (Figure 1).

## Results

### Broad cytokine responses to irradiated SARS‐CoV‐2

**Figure 2:**
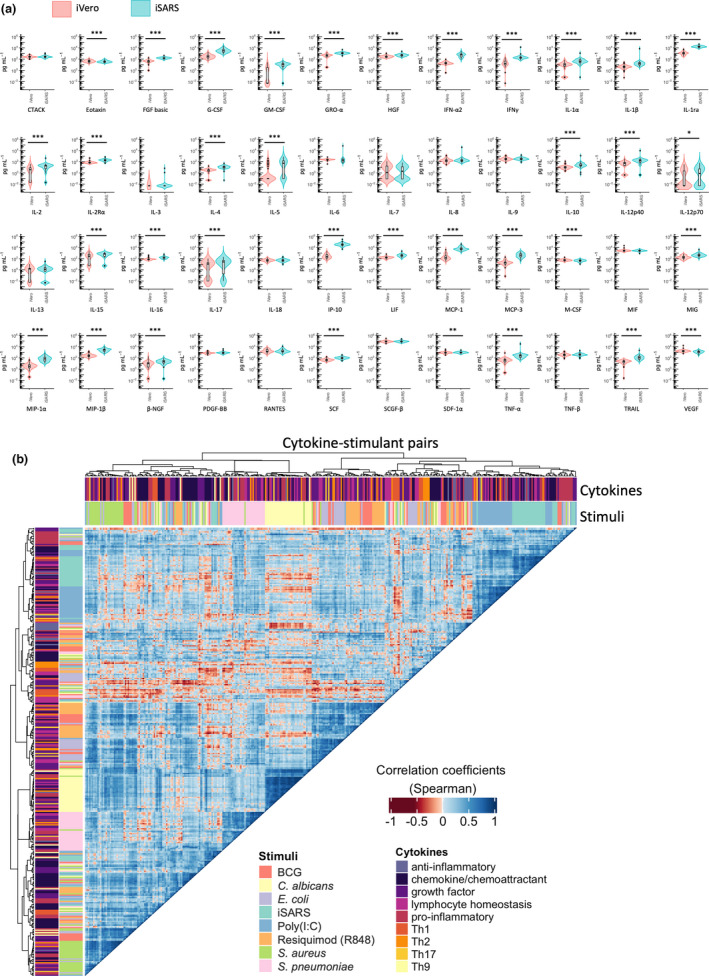
Whole‐blood cytokine responses to SARS‐CoV‐2. **(a)** Violin and Tukey boxplots of cytokine concentration (in pg mL^−1^) following stimulation of whole blood, taken prior to randomisation, with γ‐irradiated SARS‐CoV‐2 (iSARS) or γ‐irradiated uninfected Vero cell supernatant (iVero). Centre lines indicate medians; box limits indicate 25–75th percentiles; whiskers extend to 1.5 times the interquartile range from the 25th and 75th percentiles; and outliers are represented by dots. Stimulation effect measured by the Wilcoxon signed‐rank test comparing the iSARS with the iVero response (*n* = 50). Asterisks in the box plots depict significance (*P < 0.05, **P < 0.01 and ***P < 0.001). **(b)** The results from unsupervised hierarchical clustering of whole‐blood cytokine responses to a range of pathogens and TLR agonist ligands are shown. Clustering was done using Spearman’s correlation as the measure of similarity between cytokine–stimulant pairs. Red indicates a strong negative correlation, whereas blue indicates a strong positive correlation. The data shown are from samples taken prior to randomisation in participants with no missing cytokine–stimulant pair results (*n* = 38). BCG, bacillus Calmette–Guérin; iSARS, γ‐irradiated SARS‐CoV‐2; and Th, T helper.

To investigate the immune responses to SARS‐CoV‐2, we established an *in vitro* whole‐blood stimulation assay. Samples taken from BRACE trial participants prior to randomisation were stimulated for 20 h with gamma‐irradiated SARS‐CoV‐2‐infected (iSARS) and mock‐infected (iVero) Vero cell supernatants. Compared with iVero, we found significant stimulation responses to iSARS for 35 of 48 cytokines measured (Figure 2a). Stimulation effects of iSARS were strongest for interferon (IFN)‐α2 (*P* = 7.56E−10), interleukin(IL)‐1 receptor antagonist (IL‐1RA; *P* = 7.56E−10), IFN‐γ‐induced protein (IP)‐10 (CXCL10; *P* = 2.40E−09), monocyte chemoattractant protein (MCP)‐1 [chemokine (C‐C motif) ligand 2, CCL2; *P* = 1.63E−09], MCP‐3 (CCL7; *P* = 7.56E−10) and macrophage inflammatory protein‐(MIP)‐1β (CCl4; *P* = 1.11E−09).

To characterise the iSARS stimulation‐induced cytokine responses in SARS‐CoV‐2‐naïve participants, we compared the stimulation effect of iSARS with the stimulation effect of a range of killed pathogens and Toll‐like receptor (TLR) agonists previously validated in the whole‐blood assay system.^6^, ^7^ Using unsupervised clustering analysis of cytokine–stimulant pairs, we found that cytokine responses largely clustered within stimulus groups rather than by cytokine function (Figure 2b). Between pathogen groups, iSARS responses were most highly correlated with cytokine responses to poly(I:C), and least correlated with responses to bacterial stimuli (Figure 2b).

### SARS‐CoV‐2‐induced activation of myeloid lineage cells

**Figure 3:**
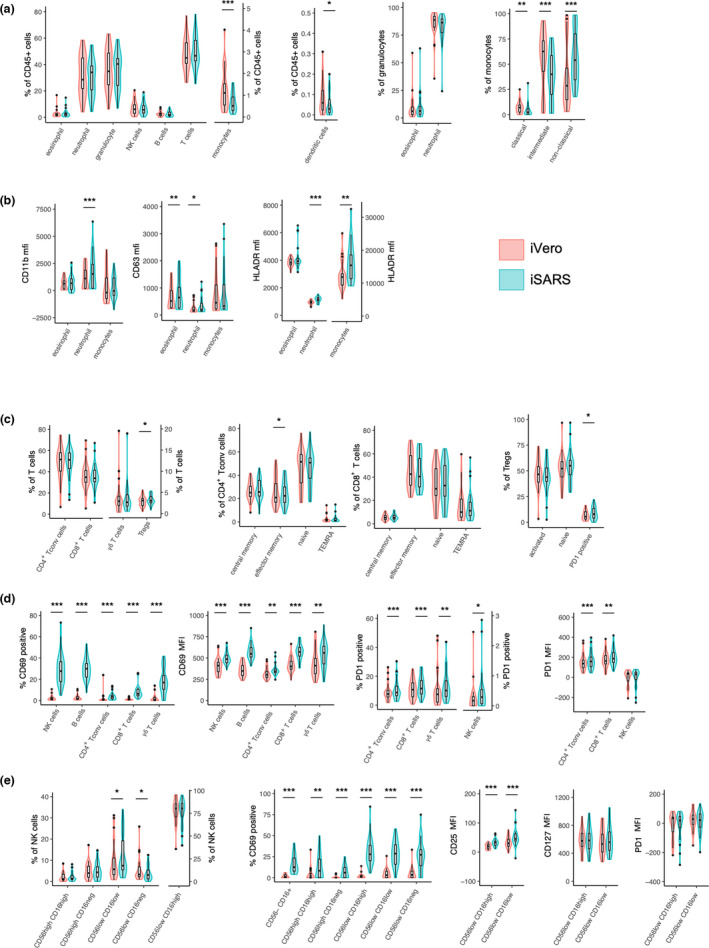
Immune cell activation in response to SARS‐CoV‐2. **(a–d)** Violin and Tukey boxplots of immune cell populations following stimulation of whole blood, taken prior to randomisation, with γ‐irradiated SARS‐CoV‐2 (iSARS) or γ‐irradiated uninfected Vero cell supernatant (iVero). Centre lines indicate medians; box limits indicate 25th–75th percentiles; whiskers extend to 1.5 times the interquartile range from the 25th and 75th percentiles; and outliers are represented by dots. Data are presented as **(a)** proportion (%) of CD45^+^, proportion of parent (granulocyte or monocyte as indicated) or expression [median fluorescence intensity (MFI)]; **(b)** proportion of total T cells or indicated T‐cell subpopulation; **(c)** proportion of CD69^+^ and PD‐1^+^ among parent populations and MFI of CD69 and PD‐1 among CD69^+^ and PD‐1^+^ cells for each parent population, respectively; and **(d)** proportion of each subset among NK cells (CD3^−^CD19^−^CD14^−^), proportion of CD69^+^ within NK cell subsets and MFI of CD25, CD127 and PD‐1 among CD56^low^CD16^high^ and CD56^low^CD16^low^ NK cells. Stimulation effect measured by the Wilcoxon signed‐rank test comparing the iSARS with the iVero response (*n* = 29). Asterisks in the box plots depict significance (*P < 0.05, **P < 0.01 and ***P < 0.001). NK, natural killer; Tconv, conventional T cell; Treg, regulatory T cell; and TEMRA, terminally differentiated effector memory T cells.

Single‐cell immunophenotyping by flow cytometry revealed activation of innate and adaptive immune cells in response to iSARS (Figure 3). Among the major immune cell populations, iSARS stimulation resulted in minor reductions in proportions of non‐adherent monocyte (*P* = 3.0E−04) and dendritic cells (*P* = 0.026) compared with iVero (Figure 3a). For non‐adherent monocytes, iSARS stimulation resulted in a shift towards non‐classical phenotype (*P* = 3.5E−06) with fewer intermediate (*P* = 6.5E−06) and classical (*P* = 0.002) monocytes, as well as increased HLA‐DR expression (*P* = 0.010) than iVero stimulation (Figure 3a,b, Supplementary figure 1a). Stimulation with iSARS increased expression of CD11b (*P* = 6.0E−05), CD63 (*P* = 2.1E−05) and HLA‐DR (*P* = 7.0E−05) on neutrophils, as well as CD63 (*P* = 0.001) on eosinophils (Figure 3b and Supplementary figure 1a).

### Activation of lymphocytes by SARS‐CoV‐2

Among T‐cell populations, iSARS stimulation increased the proportion of regulatory T cells (Tregs; *P* = 0.039) and the proportion of PD‐1^+^ Tregs (*P* = 0.013), and decreased the proportion of effector memory conventional CD4^+^ T cells (Tconv; *P* = 0.019; Figure 3c).

Analysis of activation‐induced markers revealed an increase in the proportion of CD69^+^ and the level of CD69 expression on CD69^+^ natural killer (NK; proportion, *P* = 2.6E−06; MFI, *P* = 2.0E−04), B (proportion, *P* = 2.6E−06; MFI, *P* = 2.6E−06) and γδ T (proportion, *P* = 3.0E−06; MFI, *P* = 0.005) cells with smaller increases observed for CD4^+^ Tconv (proportion, *P* = 7.3E−05; MFI, *P* = 0.003) and CD8^+^ T (proportion, *P* = 2.6E−06; MFI, *P* = 6.5E−06) cells in response to iSARS stimulation than iVero (Figure 3d and Supplementary figure 1b). Among T‐ and NK cell populations, there was also a small increase in PD‐1^+^ cells (NK cells, *P* = 0.03; CD4^+^ Tconv, *P* = 3.0E−04; CD8^+^ T cells, *P* = 0.002; and γδ T cells, *P* = 0.009) following iSARS compared with iVero stimulation, as well as an increase in PD‐1 expression on PD‐1^+^CD4^+^ Tconv (*P* = 4.0E−04) and PD‐1^+^CD8^+^ T (*P* = 0.002) cells (Figure 3d and Supplementary figure 1c).

Given the strong iSARS responses in NK cells, we further investigated the effects of iSARS on NK cell subpopulations.^8^ We observed a small decrease in CD56^low^CD16^−^ NK cells (*P* = 0.013) and an increase in CD56^low^CD16^low^ NK cells (*P* = 0.046; Figure 3e). The iSARS‐induced increase in CD69^+^ NK cells was observed across all NK cell subpopulations (CD56^low^CD16^−^, *P* = 1.5E−05; CD56^low^CD16^low^, *P* = 3.0E−06; CD56^low^CD16^high^, *P* = 2.6E−06; CD56^high^CD16^−^, *P* = 3.0E−04; and CD56^high^CD16^high^, *P* = 0.001) and a population of CD56^low^CD16^+^(CD3^−^CD19^−^CD14^−^) cells (*P* = 4.0E−04; Figure 3E). Moreover, among the predominant NK cell subpopulations, CD56^low^CD16^high^ and CD56^low^CD16^low^, we observed increased CD25 expression [CD56^low^CD16^high^ (*P* = 3.2E−06) and CD56^low^CD16^low^ (*P* = 5.0E−04)] but no changes in CD127 or PD‐1 expression (Figure 3e and Supplementary figure 1d).

### BCG vaccination‐induced reduction in cytokine responses to SARS‐CoV‐2

To investigate the effects of BCG vaccination on immune responses to SARS‐CoV‐2, we compared cytokine responses and immune cell phenotypes in paired samples taken from BRACE trial participants at baseline and 3 months after randomisation (Figure 1). The demographic details of the participants included in this analysis are presented in Table 1. Demographic details were similar between both groups for the cytokine and single‐cell immunophenotyping analyses with the exception of older age [mean 51.0 (±17.7 SD) years for BCG‐vaccinated compared with 42.1 (±15.9 SD) years for placebo‐vaccinated] and higher proportion of prior BCG vaccination (56% for BCG‐vaccinated compared with 38% for placebo‐vaccinated) in the single‐cell immunophenotyping analysis. Consistent with the low community transmission of SARS‐CoV‐2 in Australia at the time of the study, all participants were SARS‐CoV‐2 spike IgG antibody‐negative at both sample collection time points (Table 1).

**Table 1:** Demographics and other characteristics of participants

Factor	Cytokine responses		Single‐cell immunophenotyping	
	Placebo‐vaccinated *n* = 22	BCG‐vaccinated *n* = 28	Placebo‐vaccinated *n* = 8	BCG‐vaccinated *n* = 9
Age at randomisation (years), mean (SD)	39.0 (13.8)	41.4 (15.3)	42.1 (15.9)	51.0 (17.7)
Sex				
Female	18 (82%)	22 (79%)	6 (75%)	8 (89%)
Previously diagnosed/treated for latent TB				
No	21 (96%)	28 (100%)	7 (88%)	9 (100%)
Unsure	1 (5%)	0 (0%)	1 (13%)	0 (0%)
Previous positive (> 5 mm) TST				
Yes	2 (9%)	3 (11%)	2 (25%)	3 (33%)
Unsure	0 (0%)	2 (7%)	0 (0%)	0 (0%)
Previous BCG vaccination				
Yes	6 (27%)	11 (39%)	3 (38%)	5 (56%)
Comorbidities for COVID‐19^†^				
Yes	4 (18%)	5 (18%)	3 (38%)	4 (44%)
Vaccine administered perfectly				
Yes	21 (96%)	25 (89%)	7 (88%)	9 (100%)
Time (days) between randomisation and 3‐month blood, mean (SD)	103.0 (8.9)	102.3 (7.1)	96.8 (1.0)	96.1 (1.5)
Positive SARS‐CoV‐2 serology (anti‐spike)				
Baseline blood	0 (0%)	0 (0%)	0 (0%)	0 (0%)
3‐month blood	0 (0%)	0 (0%)	0 (0%)	0 (0%)

SD, standard deviation; TB, tuberculosis; TST, tuberculin skin test.

† Diabetes (any type), cardiovascular disease, chronic respiratory disease.

**Figure 4:**
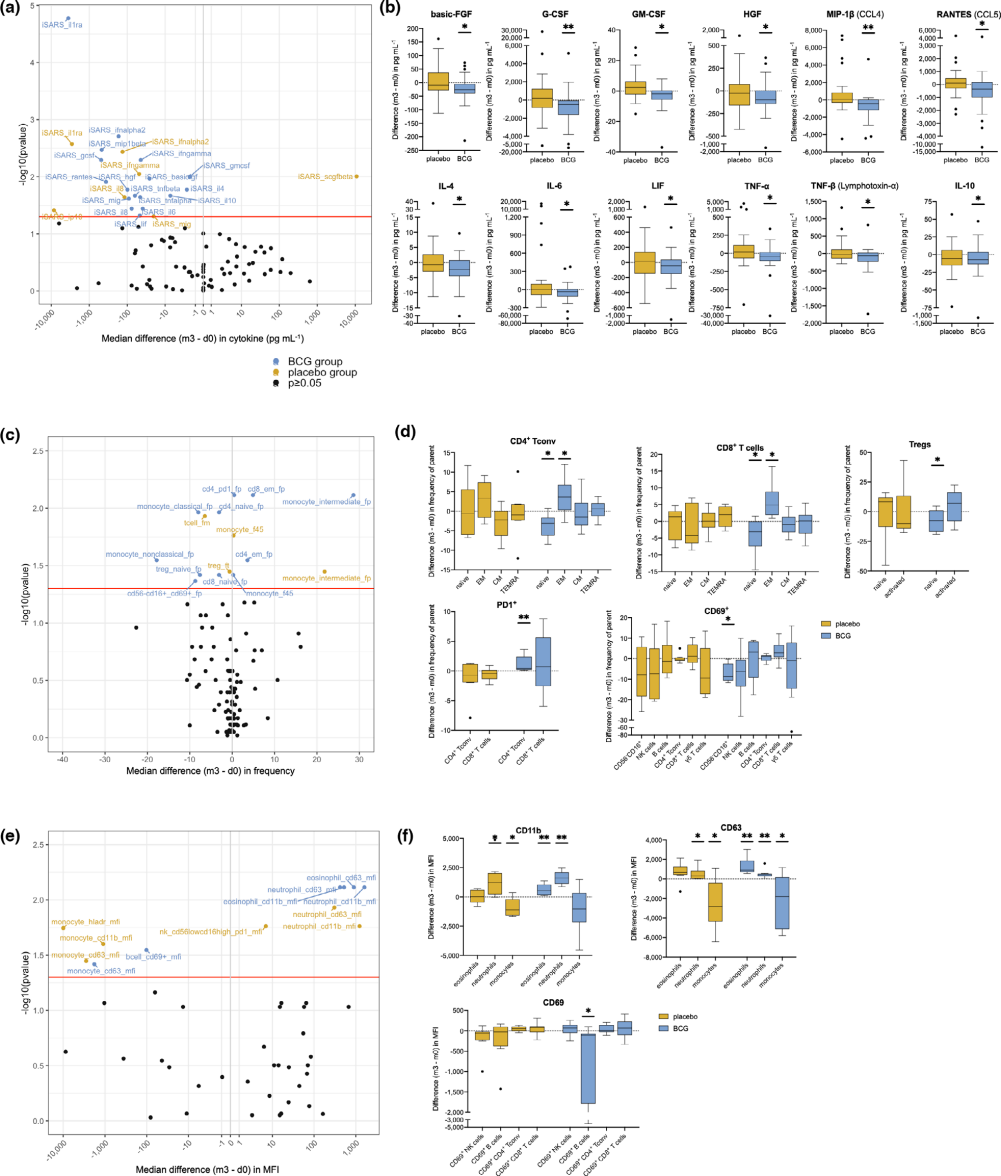
Effect of BCG vaccination on immune responses to γ‐irradiated SARS‐CoV‐2. Volcano and Tukey boxplots depicting differences in γ‐irradiated SARS‐CoV‐2 (iSARS) stimulation effect (iSARS‐iVero response) on whole‐blood **(a, b)** cytokine (*n* = 50), **(c, d)** immune cell population (*n* = 17) and **(e, f)** extracellular marker (*n* = 17) responses 3 months after randomisation. Differences in iSARS stimulation effect measured by the Wilcoxon signed‐rank test comparing the iSARS stimulation effect at 3 months (m3) post‐randomisation with the iSARS stimulation effect at baseline (d0). Volcano plots: blue dots show changes in the BCG‐vaccinated group, gold dots show changes in the placebo‐vaccinated group, and black dots show immune responses for which there was no change. Red line indicates *P* = 0.05. Boxplots: Centre lines indicate medians; box limits indicate 25–75th percentiles; whiskers extend to 1.5 times the interquartile range from the 25th and 75th percentiles; and outliers are represented by dots. Asterisks in the box plots depict significance (*P < 0.05, **P < 0.01 and ***P < 0.001). _f45, frequency of CD45^+^ cells; _fp, frequency of parent; _fm, frequency of mononuclear cells; _ft, frequency of T cells; BCG, bacillus Calmette–Guérin; CM, central memory; EM, effector memory; iSARS, γ‐irradiated SARS‐CoV‐2; iVero, γ‐irradiated uninfected Vero; MFI, median fluorescence intensity; NK, natural killer; Treg, regulatory T cell; and TEMRA, terminally differentiated effector memory T cells.

Three months following randomisation, there were changes in cytokine responses to iSARS for 17 of 48 cytokines in BCG‐vaccinated participants and for 7 of 48 in placebo‐vaccinated participants (Figure 4a; Supplementary table 1). Changes that were observed only for BCG‐vaccinated participants were reductions in basic fibroblast growth factor (FGF); granulocyte colony‐stimulating factor (G‐CSF); granulocyte–macrophage colony‐stimulating factor (GM‐CSF); hepatocyte growth factor (HGF); MIP‐1β; regulated on activation, normal T cell expressed and secreted (RANTES, CCL5); IL‐4; IL‐6; leukaemia inhibitory factor (LIF); tumor necrosis factor (TNF)‐α; and IL‐10 (Figure 4b; Supplementary table 1).

### BCG vaccination promotes SARS‐CoV‐2‐induced effector T cells in vitro

Analysis of changes in the proportions of immune cell subpopulations and activated cells in iSARS‐stimulated whole blood, 3 months after randomisation, revealed a larger number of changes following BCG vaccination (12/57 parameters) than placebo vaccination (4/57 parameters; Figure 4c; Supplementary table 2). Of the changes that were observed only for BCG‐vaccinated participants, several were within the T‐cell subpopulations, including an increase in effector memory and a decrease in naïve subset for both CD4^+^ T conv and CD8^+^ T cells (Figure 4d). In BCG‐vaccinated participants, there were an increase in the proportion of PD‐1^+^ among CD4^+^ T conv and a decrease in proportion of naïve Tregs (Figure 4d). BCG‐vaccinated participants also had an increase in CD69^+^ cells among a CD56^−^CD16^+^ (CD3^−^CD19^−^CD14^−^) population that did not occur in placebo‐vaccinated participants. Other changes in immune cell population proportions were similar for the BCG‐vaccinated and placebo‐vaccinated groups (Supplementary table 2).

### Enhanced SARS‐CoV‐2 eosinophil activation by BCG vaccination

Expression levels of activation markers were also compared before and 3 months after randomisation (Figure 4e; Supplementary table 3). Increased expression of CD11b and CD63 on eosinophils was observed only for BCG‐vaccinated participants, whereas changes in these markers on monocytes and neutrophils were similar for BCG‐vaccinated and placebo‐vaccinated participants (Figure 4f). There were also a decrease in CD69 expression among CD69^+^ B cells in BCG‐vaccinated participants (Figure 4f) and a decrease HLA‐DR expression on monocytes from placebo‐vaccinated participants (Supplementary table 3).

## Discussion

Using a specially developed *in vitro* assay, our study is the first to show off‐target effects of BCG vaccination on human cytokine and immune cell activation responses to SARS‐CoV‐2.

Epidemiological and cohort studies have reported variable findings on the effect of BCG vaccination on COVID‐19. This might be explained by the long duration (often decades) between BCG vaccination and outcome measures, as well as population differences (e.g. compliance with COVID‐19 prevention measures).^9^, ^10^, ^11^, ^12^ Preliminary findings from one RCT in elderly patients showed a reduction in combined clinical‐ and microbiological‐diagnosed COVID‐19 in BCG‐vaccinated, compared with placebo‐vaccinated, participants^13^ (preprint). The BCG vaccination‐induced immunomodulatory effects on SARS‐CoV‐2 responses in our study support the potential for BCG vaccination to have beneficial off‐target effects against COVID‐19 by dampening hyperactive inflammatory responses and enhancing protective leucocyte populations.

The immune response to SARS‐CoV‐2 infection contributes to disease pathogenesis with inflammation and cytokine storm contributing to the development of acute respiratory distress syndrome and multi‐organ dysfunction.^14^, ^15^ In our study, the effects of BCG vaccination on cytokine responses are opposite to the immunophenotypes associated with severe COVID‐19. Of the 12 cytokines found to have reduced SARS‐CoV‐2‐induced secretion following BCG vaccination in our study, nine have been associated with severe COVID‐19, namely G‐CSF,^16^, ^17^ GM‐CSF,^18^, ^19^ HGF,^18^ IL‐6,^17^, ^18^, ^20^, ^21^, ^22^, ^23^, ^24^, ^25^ IL‐10,^16^, ^17^, ^18^, ^20^, ^21^, ^22^, ^23^ LIF,^18^ MIP‐1β (CCL4),^18^ RANTES (CCL5)^18^, ^24^ and TNF‐α.^17^, ^18^, ^20^, ^24^, ^25^ MIP‐1β and RANTES (CCL5), however, have also been reported to be lower in severe than in mild cases.^21^ Therefore, the reduced SARS‐CoV‐2‐induced secretion of these cytokines following BCG vaccination suggests that BCG might protect against severe COVID‐19 by suppressing pathological cytokine responses.

Our findings of BCG‐induced enhancement of eosinophil activation in response to SARS‐CoV‐2 are consistent with a growing number of studies showing immunomodulatory effects of BCG on granulocyte populations. A recent study found that BCG vaccination in adults altered the phenotype of circulating neutrophils and eosinophils, and increased neutrophil responses to bacterial and fungal stimuli.^26^ Off‐target effects of BCG on eosinophils may have further implications given the potential protective effect of BCG against allergic disease and asthma.^27^ The contribution of eosinophils to SARS‐CoV‐2 immunity remains unclear, and many studies have focused on total granulocytes and neutrophils, although reduced eosinophils are associated with severe COVID‐19.^14^ One study, which extensively profiled granulocyte populations in COVID‐19 patients, found that failure to increase CD11b, CD24 or CD11a expression on eosinophils was associated with severe disease.^28^ A similar pattern was observed for CD63 expression although this was not statistically significant.^28^ These may represent a subset of activated eosinophils that are protective in COVID‐19, and the boosting of CD11b and CD63 upregulation by BCG might thus enhance this protective effect.

The role of T cells in severe COVID‐19 remains unclear. There are varying reports of CD4^+^ and CD8^+^ T‐cell exhaustion, and both expansion and depletion of effector/activated/terminally differentiated T cells in severe COVID‐19.^15^, ^29^ T‐cell production of the inflammatory cytokines IL‐6, GM‐CSF and GM‐CSF/IFN‐y is associated with severe COVID‐19.^30^, ^31^ In the context of COVID‐19, CD4^+^ and CD8^+^ TEMRA are the major sources of GM‐CSF associated with disease severity. In contrast, T‐cell lymphopenia is a common feature of severe COVID‐19, and T cells have been inversely correlated with cytokines prominent in severe COVID‐19.^15^, ^29^ Studies have shown reduced proportions or numbers of CD4^+^ and CD8^+^ effector memory and TEMRA, and reduced numbers of CD4^+^ and CD8^+^ naïve, central memory, effector memory and TEMRA in severe COVID‐19.^18^, ^32^ These reductions are evident within the first 12 days and persist longest in patients with the most severe severity.^32^ We found that BCG vaccination increased effector memory and decreased naïve T cells and Treg proportions following SARS‐CoV‐2 stimulation. Together with the decreased GM‐CSF and IL‐6 responses observed in our study, our findings suggest that rapid promotion of effector memory T cells and reduction in inflammatory cytokine responses by BCG vaccination might protect against the development of severe COVID‐19.

BCG off‐target effects on innate immune populations have been linked to induction of trained immunity; however, few studies have investigated off‐target effects of BCG on T cells and the underlying mechanism.^33^ A recent study showed that *in vitro* exposure to BCG‐derived peptides and BCG vaccination both boosted CD4^+^ and CD8^+^ T‐cell responses to SARS‐CoV‐2 peptides.^34^ This suggests that one mechanism underpinning the enhanced effector T‐cell responses to SARS‐CoV‐2 in our study could be cross‐reactivity between BCG and SARS‐CoV‐2 antigens. However, the lack of association between levels of SARS‐CoV‐2 antigen‐specific T‐cell IFN‐γ responses in mild COVID‐19 despite increased effector CD8^+^ T cells, and the enrichment of TCR‐activated pathways in severe COVID‐19, also suggests a role for bystander activation.^32^

In transgenic mice expressing the human ACE2 receptor, BCG vaccination is partially protective against a lethal SARS‐CoV‐2 challenge. In two studies, prior subcutaneous BCG vaccination reduced SARS‐CoV‐2 infection‐induced weight loss but did not alter overall disease score.^35^, ^36^ Prior BCG vaccination also reduced SARS‐CoV‐2 infection‐induced IL‐6, CXCL‐1 (IL‐8 equivalent in mice) and GM‐CSF in lung tissue.^35^, ^36^ In one model, co‐administration of BCG with SARS‐CoV‐2 spike and SARS‐CoV‐2 spike+alum vaccination boosted immunity with increased CD4^+^ and CD8^+^ T‐cell responses to a greater extent than SARS‐CoV‐2 spike or SARS‐CoV‐2 spike+alum vaccines alone.^35^ Moreover, concomitant BGG with the SARS‐CoV‐2 spike+alum completely protected against lethal SARS‐CoV‐2 infection in the humanised ACE2 receptor mice.^35^ There are limitations to these murine models, in that BCG vaccination causes infection in mice, the route of administration differs (intradermal injection in humans compared with subcutaneous and intravenous injections in these models), wild‐type mice are resistant to SARS‐CoV‐2, and there is rapid onset of severe symptoms in the SARS‐CoV‐2‐infected transgenic mice. Despite this, the decreased inflammatory cytokine responses and increased T‐cell responses observed in our study are consistent with the findings from these models. This suggests that, in addition to protecting against COVID‐19 directly, BCG vaccination might also boost the efficacy of COVID‐19‐specific vaccines.

The stimulation model developed in this study has significant implications for future research on SARS‐CoV‐2. In this model, short‐term *in vitro* stimulation with SARS‐CoV‐2 induced a range of innate and adaptive immune cell populations and secretion of a broad panel of cytokines, chemokines and growth factors. The development of this model for *in vitro* stimulation using inactivated virus enables analyses of immune responses to SARS‐CoV‐2 in a diverse range of laboratory settings. There are multiple methods for virus inactivation; however, common methods, such as chemical and heat inactivation, can damage structural proteins and viral antigens.^37^ In contrast, γ‐irradiation can effectively inactivate a range of viruses^38^ including SARS‐CoV‐2^39^ whilst maintaining immunoreactivity.^40^ The potent γ‐irradiated SARS‐CoV‐2‐induced activation of immune cells from SARS‐CoV‐2‐naïve adults in our assays supports the potential of γ‐irradiated SARS‐CoV‐2 vaccines^41^, ^42^ and provides a model for future studies assessing modulation of immune responses to SARS‐CoV‐2.

In SARS‐CoV‐2‐unexposed adults, we found that cytokine responses to a range of inactivated pathogens and TLR agonists were more strongly correlated within stimulation groups rather than by cytokine. This is consistent with previous studies of *in vitro* cytokine responses, which also show organisation of cytokine responses within pathogen groups rather than by immune response pathways.^43^, ^44^ The close association between responses to iSARS and poly(I:C) in our study suggests a role for the TLR3 pathway in immune responses to SARS‐CoV‐2. Multiple TLRs have been associated with immune responses to SARS‐CoV‐2. TLR3 recognises double‐stranded RNA and is activated by single‐stranded RNA viruses as they generate double‐stranded RNA during the replication process. Low TLR3 expression has been linked to severe COVID‐19,^45^ and the TLR3 pathway has been shown to be protective against the related SARS‐CoV in murine models.^46^ Moreover, TLR3 has been shown to be an early driver of cytokine responses to SARS‐CoV‐2 with a later contribution by TLR7.^47^ Together, these data support a role for TLR3 in the early innate immune response to SARS‐CoV‐2 infection.

The limitations of our study include the low participant number for the single‐cell immunophenotyping analysis. However, the impact of this was mitigated by the inclusion of samples from placebo‐vaccinated participants, which ensured that changes resulting from environmental and technical variance over time could be detected. Additionally, we did not correct for potential confounders, although key factors (i.e. sex, comorbidities) were similar between the BCG‐vaccinated and placebo‐vaccinated groups. However, because of the small sample size in the single‐cell immunophenotyping analysis, we were unable to exclude potential confounding by the small difference between the groups in age and prior BCG vaccination status. We were unable to determine whether the BCG‐induced changes in SARS‐CoV‐2 responses observed in our study are associated with protection against severe COVID‐19 as SARS‐CoV‐2 community transmission in the study setting (Melbourne, Australia) was low over the 12‐month follow‐up period of these participants in the BRACE trial.

In conclusion, we found that BCG vaccination has off‐target effects on innate and adaptive immune responses to SARS‐CoV‐2. The immunomodulatory signature of BCG’s off‐target effects on SARS‐CoV‐2 responses is distinct from that observed for bacterial and fungal stimuli. Moreover, the BCG‐induced changes in SARS‐CoV‐2 immunity are in direct contrast to immune signatures of severe COVID‐19. These findings provide support for the hypothesis that BCG vaccination might reduce the severity of COVID‐19.

## Methods

### Subjects and study design

Participants were a subset of participants from BRACE trial sites in Victoria, Australia.^5^ The inclusion and exclusion criteria for the BRACE trial have been previously described.^5^ Participants were randomised to receive 0.1 mL intradermal vaccination with BCG‐Denmark (AJ Vaccines, Copenhagen, Denmark; BCG‐vaccinated) or NaCl (placebo‐vaccinated). At randomisation and the 3‐month study visit, venous blood was collected from participants into a lithium heparin tube (Greiner Bio‐One, Frickenhausen, Germany) and, in a subset set of participants, also into a sodium heparin tube (BD, Plymouth, UK). The latter were eligible for inclusion in this immunological substudy if they had a sodium heparin whole‐blood sample stimulated with iSARS. This stimulation was done for all participants whose blood sample was collected at an appropriate date and time for iSARS stimulation, with a sufficient volume of blood collected in a sodium heparin tube available. Participants were excluded from the substudy if they had a positive SARS‐CoV‐2 test result between randomisation and the 3‐month blood sample.

### Preparation of stimuli

The SARS‐CoV‐2 [hCoV‐19/Australia/VIC01/2020 (VIC01, NCBI: MT007544.1)] virus was kindly gifted to us by the Victorian Infectious Diseases Reference Laboratory (VIDRL). Virus stocks were prepared by growing VIC01 in Vero cells in serum‐free MEM in the presence of 1 µg mL^−1^ TPCK Trypsin (Worthington Biochemical Corporation, Lakewood, USA). Serum‐free media contained MEM (from Media Preparation Unit, Peter Doherty Institute), 5% FBS (Sigma‐Aldrich, St. Louis, USA), 50 U mL^−1^ penicillin and 50 µg mL^−1^ streptomycin (Gibco, Bleiswijk, The Netherlands), 2 mM GlutaMAX (Gibco) and 15 mm HEPES (Gibco). Gamma irradiation of SARS‐CoV‐2‐infected (iSARS) and mock‐infected (iVero) supernatants was performed by Steritech at a dose of 50 kGy. Viral titres were determined in pre‐ and post‐gamma irradiation samples to confirm virus inactivation.

Preparation of other stimuli was described previously.^6^ The final concentrations of stimuli in the assay strips were as follows: RPMI, 1:2; iVero, 1:10; iSARS, 10^5.2^ TCID_50_ mL^−1^; BCG‐Denmark (Serum Statens Institut), 75 µg mL^−1^; heat‐killed *Candida albicans*, 1.0 × 10^6^ CFU mL^−1^; heat‐killed *Escherichia coli*, 1.0 × 10^6^ CFU mL^−1^; heat‐killed *Staphylococcus aureus*, 1.0 × 10^7^ CFU mL^−1^; heat‐killed *Streptococcus pneumoniae* serotype 15C (non‐vaccine serotype), 1.0 × 10^7^ CFU mL^−1^; poly(I:C; high‐molecular weight), 5 µg mL^−1^; and resiquimod (R848), 3 µg mL^−1^.

### Whole‐blood stimulation

Whole blood, diluted 1:1 with RPMI 1640 medium (GlutaMAX Supplement, HEPES, Gibco, Life Technologies, Bleiswijk, The Netherlands), was added to pre‐prepared stimulation assay strips. When insufficient blood was available for all stimulations, a predetermined priority order was used. Whole blood was stimulated at 37°C (5% CO_2_) for 20 (±2) hours. Following stimulation, supernatants were harvested and stored at −80°C for future cytokine analysis and cells were immediately prepared for single‐cell immunophenotyping.

### Single‐cell immunophenotyping

Following stimulation and removal of the supernatant, red blood cells (RBCs) were lysed by incubation in RBC lysis buffer for 10 min. The cells were washed twice in PBS and resuspended in 200 µl PBS; then, 100 µl was transferred each into two wells of a 96‐well plate. Fixable viability stain LIVE/DEAD™ Fixable Near‐IR Dead Cell Stain (Panel 1, Invitrogen, Eugene, USA) or fixable viability stain BV510 (Panel 2, BD biosciences, New Jersey, USA) was used with a final concentration of 1 µl mL^−1^. Cells were incubated for 15 min [light‐protected, at room temperature prior to centrifugation and washing in FACS buffer (2% FCS, 2 mm EDTA in PBS)]. Samples were resuspended in 20 μL FACS buffer containing human FC‐block (BD Biosciences) and incubated (light‐protected) for 5 min at room temperature. The following markers (BD Biosciences) were included in the antibody cocktails: Panel 1: CD3, CD4, CD8, CD11b, CD11c, CD14, CD15, CD16, CD19, CD45, CD56, CD63 and HLA‐DR; and Panel 2: CCR7, CD3, CD4, CD8, CD11c, CD127, CD14, CD16, CD19, CD25, CD45RA, CD56, CD69, HLA‐DR, PD‐1 and γδ TCR. Twofold concentrated antibody cocktails were added 1:1 to cells, then incubated (light‐protected) for 30 min on ice. Cells were then washed in FACS buffer prior to fixation in 2% PFA for 20 min on ice (light‐protected). Fixed cells were washed and resuspended in FACS buffer for acquisition using a LSR II X‐20 Fortessa (BD). Compensation was made at the time of acquisition using a combination of single‐stained cells and compensation beads (BD Biosciences).

### Cytokine analysis

Supernatants from stimulated whole blood were diluted 1:5 prior to quantification of secreted cytokines, chemokines and growth factors using the Bio‐Plex Pro Human Cytokine 48‐Plex Screening Panel (Bio‐Rad, California, USA) according to the manufacturer’s instructions. The following analytes were measured: CTACK, Eotaxin, Basic FGF, G‐CSF, GM‐CSF, GRO‐α, HGF, IFN‐α2, IFN‐γ, IL‐1α, IL‐1β, IL‐1rα, IL‐2, IL‐2rα, IL‐3, IL‐4, IL‐5, IL‐6, IL‐7, IL‐8, IL‐9, IL‐10, IL‐12(p40), IL‐12(p70), IL‐13, IL‐15, IL‐16, IL‐17, IL‐18, IP‐10, LIF, MCP‐1, MCP‐3, M‐CSF, MIF, MIG, MIP‐1α, MIP‐1β, β‐NGF, PDGF‐BB, RANTES, SCF, SCGF‐β, SDF‐1α, TNF‐α, TNF‐β, TRAIL and VEGF. Data were acquired on the Bio‐Plex 200 system using the Bio‐Plex Manager™ 6.1 software (Bio‐Rad).

### SARS‐CoV‐2 anti‐spike ELISA

Plasma was isolated from whole blood collected in lithium heparin tubes and stored at −80°C until analysis. Anti‐SARS‐CoV‐2 antibodies in plasma samples were detected using Wantai SARS‐CoV‐2 Total Ab ELISA (Beijing Wantai Biological Pharmacy Enterprise, Beijing, China) as previously described.^48^

### Data analysis

For all sample collection, laboratory processing and analysis, data cleaning and data processing, researchers were blinded to the BCG vaccination status of participants.

#### Cytokine analysis

As per our previous studies, values below the lower limit detection for each analyte were assigned a value of half the lowest detected value for that analyte. Only participants with matched baseline (pre‐randomisation) and 3‐month samples available were included in the cytokine data analysis. Data were non‐parametric before and after log transformation as determined by a Shapiro–Wilk normality test using *P* < 0.05 as the threshold for non‐normal distribution (data not shown); therefore, non‐parametric tests were used on non‐transformed data for all analyses.

#### Correlation analysis

Unsupervised hierarchical clustering of cytokine–stimulant pairs was done in the statistical programming language R using R Studio.^49^ Cytokine–stimulant pairs with > 10% missing data were excluded (BCG: IL‐1β, IL‐6, IL‐8, MCP‐1, MIP‐1α and MIP‐1β; *C. albicans*: IL‐8, MCP‐1 and MIP‐1α; *E. coli*: IL‐6, MCP‐1, MIP‐1α and MIP‐1β; *S. aureus*: IL‐8, MCP‐1, MIP‐1α and MIP‐1β; *S. pneumoniae*: IL‐6, IL‐8, MCP‐1, MIP‐1α and MIP‐1β; and R848: IL‐1β, IL‐6, MIP‐1α and MIP‐1β). Spearman’s correlation was the measure of similarity used, and clustering was done using these coefficients as distance by an unsupervised hierarchical clustering approach.

#### Single‐cell immunophenotyping analysis

Flow cytometry files were preprocessed using FlowJo version 10.7.1 (FlowJo, Oregon, USA). Debris, doublets, RBC and non‐live cells were removed using standard preprocessing. Where required, compensation was adjusted at the time of analysis. Supplementary figure 2 depicts the manual gating strategy for each of the panels. For MFI analysis, immune cell subsets with less than 20 cells in the subset for at least 50% of the samples were excluded from MFI analysis. This resulted in the exclusion of dendritic cells, and CD56^high^CD16^−^, CD56^high^cd15^high^ and CD56^low^CD16^−^ NK cells from MFI analysis. Only participants with matched baseline (pre‐randomisation) and 3‐month samples were included in single‐cell immunophenotyping analysis of differences in iSARS stimulation effect after randomisation.

#### Statistical analysis

Stimulation effect of iSARS in baseline (pre‐randomisation) samples was determined by a Wilcoxon signed‐rank test comparing the iSARS with the iVero response in paired samples. Differences in iSARS stimulation effect after randomisation were measured by a Wilcoxon signed‐rank test comparing the iSARS stimulation effect (iSARS response minus iVero response) at 3 months post‐randomisation with the iSARS stimulation effect at baseline in paired samples. This was done separately for participants in the BCG vaccination and placebo‐vaccinated groups. As these analyses were exploratory, we did not adjust for multiple testing. Statistical analysis was performed using Stata (version 13.1; College Station, USA) and depicted graphically using R and GraphPad Prism (version 9.1.0; California, USA).

## Funding

The Murdoch Children’s Research Institute (MCRI) leads the BRACE trial across 36 sites in five countries. It is supported by the Victorian Government’s Operational Infrastructure Support Programme. The BRACE trial is supported by the Bill & Melinda Gates Foundation (INV‐017302), the Minderoo Foundation (COV‐001), Sarah and Lachlan Murdoch, the Royal Children's Hospital Foundation (2020‐1263 BRACE Trial), Health Services Union NSW, the Peter Sowerby Foundation, the Ministry of Health Government of South Australia, the NAB Foundation, the Calvert‐Jones Foundation, the Modara Pines Charitable Foundation, the UHG Foundation Pty Ltd, Epworth Healthcare and individual donors. NC is supported by the National Health and Medical Research Council (NHMRC; Investigator Grant GNT1197117). LFP is supported by the Swiss National Science Foundation (Early Postdoc. Mobility grant number P2GEP3_178155). The Melbourne WHO Collaborating Centre for Reference and Research on Influenza is supported by the Australian Government Department of Health. KS is supported by an NHMRC Investigator grant and has received support from the Victorian Government Department of Health, Jack Ma Foundation and the A2 Milk Company.

## Conflict of interest

The authors declare that they have no relevant conflict of interest.

## Author contribution

**Nicole L Messina:** Conceptualization; Data curation; Formal analysis; Funding acquisition; Investigation; Methodology; Project administration; Visualization; Writing – original draft; Writing – review & editing. **Susie Germano:** Conceptualization; Data curation; Methodology; Project administration; Supervision; Writing – review & editing. **Rebecca McElroy:** Data curation; Formal analysis; Project administration; Visualization; Writing – review & editing. **Rajeev Rudraraju:** Data curation; Methodology; Resources; Validation; Writing – review & editing. **Rhian Bonnici:** Data curation; Project administration; Writing – review & editing. **Laure F Pittet:** Formal analysis; Investigation; Visualization; Writing – review & editing. **Melanie R Neeland:** Methodology; Resources; Visualization; Writing – review & editing. **Suellen Nicholson:** Data curation; Methodology; Resources; Writing – review & editing. **Kanta Subbarao:** Conceptualization; Methodology; Resources; Supervision; Writing – review & editing. **Nigel Curtis:** Conceptualization; Funding acquisition; Methodology; Resources; Supervision; Writing – review & editing.

## Ethical approval

The BRACE trial was approved by the Royal Children’s Hospital Human Research Ethics Committee (No. 62586) with HREC and/or governance approval at all participating sites.

## Supporting information

Supplementary figure 1. Representative histograms of iSARS stimulation effect.
Supplementary figure 2. Single cell immunophenotyping manual gating strategies.
Supplementary table 1. Difference (3‐months ‐ baseline) in stimulation effect on cytokine responses
Supplementary table 2. Difference (3‐months ‐ baseline) in stimulation effect on immune cell populations
Supplementary table 3. Difference (3‐months ‐ baseline) in stimulation effect on immune cell marker expression levels
