# Supplementary material for: Off‐target effects of bacillus Calmette–Guérin vaccination on immune responses to SARS‐CoV‐2: implications for protection against severe COVID‐19
Source: Clin Transl Immunology. 2022 Apr 22;11(4):e1387. doi: 10.1002/cti2.1387 (PMC9028103; doi:10.1002/cti2.1387)
Supplement: Supplementary file 1 — Supplementary figure 1. Representative histograms of iSARS stimulation effect. Supplementary figure 2. Single cell immunophenotyping manual gating strategies. Supplementary table 1. Difference (3‐months ‐ baseline) in stimulation effect on cytokine responses Supplementary table 2. Difference (3‐months ‐ baseline) in stimulation effect on immune cell populations Supplementary table 3. Difference (3‐months ‐ baseline) in stimulation effect on immune cell marker expression levels [file CTI2-11-e1387-s001.pdf]

Off-target effects of BCG vaccination on immune responses to SARS-CoV-2: implications for protection against severe COVID-19

Nicole L Messina, Susie Germano, Rebecca Mcelroy, Rajeev Rudraraju, Rhian Bonnici, Laure F Pittet, Melanie R Neeland, Suellen Nicholson, Kanta Subbarao, Nigel Curtis, the BRACE trial Consortium Group

Supporting information

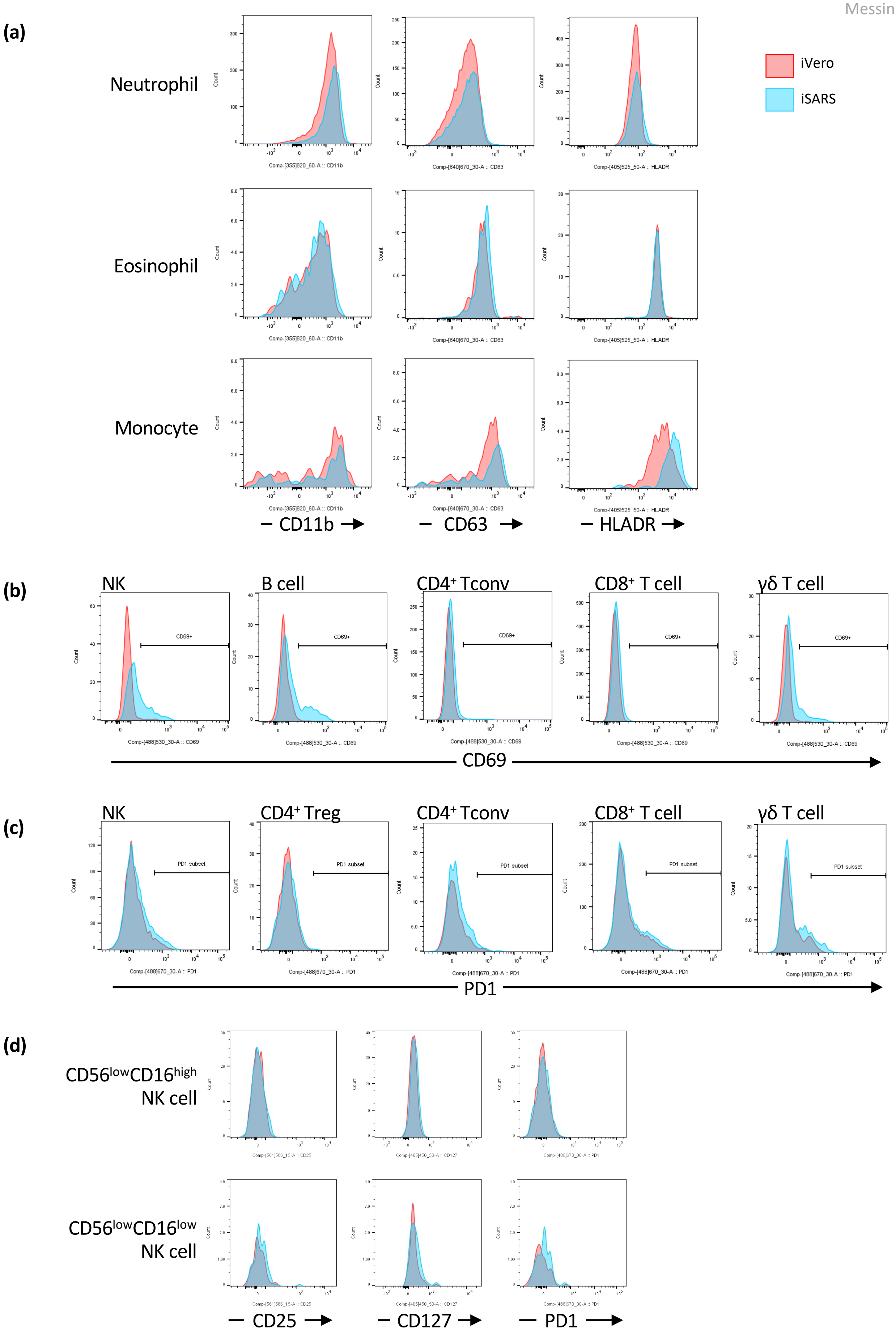

Supplementary figure 1: Representative histograms of iSARS stimulation effect.
Overlay histograms of one representative sample showing cell surface marker expression on immune cell subpopulations in iVero (red) and iSARS (blue) stimulated whole blood samples.

Supplementary table 1: Difference (3-months - baseline) in stimulation effect on cytokine responses

	BCG group				Placebo group			
Cytokine	N	median	IQR	*P*-value	N	median	IQR	*P*-value
CTACK	28	6.6	96.7	0.767	22	33.1	133.1	0.168
Eotaxin	28	-1.8	14.7	0.466	22	0.1	9.7	0.733
FGF basic	28	-25.9	33.5	**0.011**	22	-9.3	59.5	0.833
G-CSF	28	-485.1	1490.6	**0.005**	22	199.0	1881.9	0.485
GM-CSF	28	-1.8	5.7	**0.010**	22	2.4	8.2	0.101
GRO-α	28	-135.1	492.4	0.080	22	119.9	353.6	0.277
HGF	28	-99.0	142.7	**0.017**	22	-25.1	217.6	0.833
IFN-α2	28	-169.2	432.0	**0.002**	22	-133.5	431.9	**0.004**
IFNγ	28	-44.2	85.5	**0.005**	22	-48.8	66.4	**0.009**
IL-1α	28	-7.3	31.9	0.172	22	7.6	38.0	0.249
IL-1β	28	-2.7	12.0	0.080	22	0.7	13.3	0.808
IL-1ra	28	-3558.3	5051.0	**0.000**	22	-2861.0	5735.6	**0.003**
IL-2	28	1.1	14.4	0.847	22	0.0	15.2	0.346
IL-2Rα	28	-2.8	83.5	0.387	22	16.0	77.5	0.338
IL-3	28	0.0	0.0	0.979	22	0.0	0.0	0.974
IL-4	28	-2.3	4.7	**0.017**	22	-0.7	4.5	0.527
IL-5	28	0.0	45.7	0.419	22	12.2	120.4	0.170
IL-6	28	-39.1	121.0	**0.036**	22	2.8	153.2	0.961
IL-7	28	0.0	5.4	0.876	22	0.0	21.4	0.099
IL-8	28	-76.5	238.8	**0.036**	21	-118.3	354.7	**0.023**
IL-9	28	-50.8	198.3	0.076	22	-23.0	211.9	0.910
IL-10	28	-7.2	17.6	**0.021**	22	-5.3	18.0	0.140
IL-12p40	28	-23.5	110.8	0.158	22	23.5	101.2	0.205
IL-12p70	28	0.0	3.2	0.567	22	0.0	6.4	0.375
IL-13	28	0.0	2.6	0.465	22	0.4	2.1	0.167
IL-15	28	-3.3	468.5	0.973	22	60.4	615.9	0.178
IL-16	28	-10.8	49.3	0.133	22	4.9	54.5	0.485
IL-17	28	0.0	22.3	0.954	22	-1.5	26.4	0.548
IL-18	28	7.1	19.5	0.151	22	4.6	21.3	0.123
IP-10	26	-6236.7	26968.5	0.066	21	-8480.9	28894.0	**0.039**
LIF	28	-46.5	182.2	**0.048**	22	16.1	273.7	0.661
MCP-1	26	-569.1	4282.3	0.732	18	637.8	4187.8	0.679
MCP-3	28	11.7	436.2	0.495	22	41.6	410.8	0.506
M-CSF	28	-2.0	18.0	0.466	22	0.9	15.6	0.638
MIF	28	-76.7	786.6	0.802	22	-56.0	543.2	0.910
MIG	28	-90.8	163.1	**0.024**	22	-20.2	153.9	**0.050**
MIP-1α	27	-6.3	61.1	0.118	20	3.3	52.6	0.502
MIP-1β	27	-470.4	1211.6	**0.003**	22	59.7	910.6	0.426
β-NGF	28	-5.0	24.0	0.374	22	2.8	24.7	0.408
PDGF-BB	28	-60.0	262.1	0.194	22	40.7	253.0	0.211
RANTES	28	-362.4	1152.7	**0.012**	22	107.6	724.3	0.465
SCF	28	-12.2	37.5	0.127	22	4.2	45.5	0.306
SCGF-β	28	-2100.2	36701.4	0.891	22	10848.1	22242.7	**0.010**
SDF-1α	28	-6.1	261.0	0.927	22	-26.2	167.1	0.638
TNF-α	28	-45.7	119.4	**0.023**	22	20.2	138.4	0.322
TNF-β	28	-62.9	246.6	**0.021**	22	-30.1	249.5	0.758
TRAIL	28	-7.2	83.5	0.116	22	7.3	105.9	0.372
VEGF	28	-62.9	531.9	0.219	22	-39.0	1086.8	0.372

SE = stimulation effect (iSARS-iVERO)

Supplementary table 2: Difference (3-months - baseline) in stimulation effect on immune cell populations

		BCG group				Placebo group			
Parameter	Frequency of	N	median	IQR	*P*-value	N	median	IQR	*P*-value
B cells	CD45+	9	-0.5	1.3	0.314	8	0.1	0.5	0.263
CD4+ (total)	CD45+	9	-3.9	6.4	0.314	8	-1.1	3.7	0.401
CD8+ T cells	CD45+	9	-1.0	1.2	0.086	8	-0.7	5.2	0.889
Dendritic cells	CD45+	9	0.0	0.1	0.515	8	0.0	0.1	0.484
Eosinophils	CD45+	9	-0.7	1.7	0.859	8	0.1	1.2	0.674
Granulocytes	CD45+	9	5.0	12.7	0.214	8	-1.0	13.2	0.674
Monocytes	CD45+	9	0.2	0.7	**0.038**	8	0.3	0.5	**0.017**
Mononuclear cells	CD45+	9	-7.1	7.0	0.110	8	-1.4	12.6	0.889
Neutrophils	CD45+	9	5.3	9.8	0.173	8	0.2	14.5	0.779
NK cells cells	CD45+	9	-0.6	0.8	0.260	8	-0.6	3.5	0.889
Non conventional T cells	CD45+	9	-1.1	1.8	**0.038**	8	-1.5	1.9	0.093
T cells	CD45+	9	-4.7	6.4	0.110	8	-4.4	5.9	0.161
B cells	Mononuclear cells	9	0.4	4.5	0.678	8	0.1	0.9	0.779
CD56-CD16+	Mononuclear cells	9	0.1	1.3	0.374	8	0.4	1.2	0.401
Dendritic cells	Mononuclear cells	9	0.0	0.1	0.260	8	0.0	0.2	0.889
Monocytes	Mononuclear cells	9	0.7	2.8	0.374	8	-0.1	1.5	0.674
NK cells	Mononuclear cells	9	-0.5	1.6	0.260	8	-0.2	0.9	0.362
T cells	Mononuclear cells	9	-10.7	9.2	0.314	8	-6.5	11.4	**0.012**
Classical	Monocytes	9	-8.0	8.6	**0.011**	8	-4.9	18.6	0.208
Intermediate	Monocytes	9	28.6	15.7	**0.008**	8	21.8	25.1	**0.036**
Non Classical	Monocytes	9	-17.8	21.4	**0.028**	8	-10.6	22.4	0.069
CD4+ Tconv	T cells	9	-3.1	6.0	0.515	8	-1.2	6.0	0.208
CD8+ T cells	T cells	9	1.0	12.9	0.767	8	1.8	6.7	0.575
Non conventional T cells	T cells	9	0.3	7.7	0.515	8	1.9	5.1	0.484
Tregs	T cells	9	-0.1	0.7	0.859	8	-0.6	0.6	**0.036**
γδ T cells	T cells	9	0.1	1.0	0.767	8	0.1	0.7	0.401
CD69+	B cells	9	3.2	13.2	0.767	8	-1.4	11.8	0.889
CD69+	CD4+ Tconv	9	1.1	1.9	0.441	8	-0.6	1.2	0.401
CD69+	CD56-CD16+	5	-8.7	5.1	**0.043**	5	-7.8	11.8	0.345
CD69+	CD8+ T cells	9	2.9	4.7	0.066	8	1.1	6.9	0.327
CD69+	NK cells	9	-6.2	5.7	0.086	8	-7.4	22.7	0.161
CD69+	γδ T cells	9	-1.0	14.3	0.678	8	-9.5	19.9	0.161
PD1+	CD4+ Tconv	9	0.5	2.0	**0.008**	8	-0.7	3.0	0.263
PD1+	CD8+ T cells	9	0.7	4.9	0.515	8	-0.4	1.4	0.263
PD1+	NK cells	9	-0.4	1.1	0.139	8	0.3	1.2	0.889
PD1+	Tregs	9	5.4	12.5	0.374	8	1.3	9.3	0.674
PD1+	γδ T cells	9	-0.3	10.5	0.594	8	-2.7	13.5	0.401
Naïve	CD4+ Tconv	9	-3.1	4.0	**0.011**	8	-0.6	11.1	0.889
Effector memory	CD4+ Tconv	9	3.6	1.2	**0.028**	8	3.3	8.2	0.123
Central memory	CD4+ Tconv	9	-1.5	3.4	0.594	8	-2.3	5.6	0.069
TEMRA	CD4+ Tconv	9	0.6	2.8	0.374	8	-1.0	4.0	0.575
Naïve	CD8+ T cells	9	-3.1	5.2	**0.038**	8	1.4	6.9	0.779
Effector memory	CD8+ T cells	9	4.9	3.0	**0.008**	8	-4.2	11.4	0.575
Central memory	CD8+ T cells	9	-1.0	3.4	0.260	8	0.1	4.3	0.779
TEMRA	CD8+ T cells	9	0.1	4.2	0.767	8	2.0	4.3	0.327
Naïve	Tregs	9	-7.6	13.1	**0.038**	8	8.4	22.4	0.674
Activated	Tregs	9	7.0	20.1	0.173	8	-10.0	25.3	0.779
CD56hiCD16-	NK cells	9	-1.1	5.6	0.953	8	2.7	5.5	0.779
CD56hiCD16hi	NK cells	9	0.9	0.9	0.173	8	0.3	3.1	0.674
CD56loCD16-	NK cells	9	0.5	2.2	0.767	8	-0.6	5.0	0.674
CD56loCD16hi	NK cells	9	1.2	11.8	0.953	8	0.5	7.5	0.624
CD56loCD16lo	NK cells	9	-0.5	12.1	0.767	8	-1.2	8.6	0.484
CD69+	CD56hiCD16- NK cells	9	1.2	8.3	0.282	8	-3.5	18.7	0.889
CD69+	CD56hiCD16hi NK cells	9	-10.0	16.7	0.282	8	-8.4	40.0	0.361
CD69+	CD56loCD16- NK cells	9	-22.6	41.1	0.110	8	0.4	29.1	0.889
CD69+	CD56loCD16hi NK cells	9	-7.4	18.5	0.086	8	-8.9	24.6	0.208
CD69+	CD56loCD16lo NK cells	9	-0.2	21.3	0.767	8	-6.3	25.8	0.263

SE = stimulation effect (iSARS-iVERO)

Supplementary table 3: Difference (3-months - baseline) in stimulation effect on immune cell marker expression levels

		BCG group				Placebo group			
MFI	Parent population	N	median	IQR	*P*-value	N	median	IQR	*P*-value
CD69	CD69+ B cells	9	-102.0	138.7	**0.028**	8	-29.0	381.0	0.327
CD69	CD69+ CD4+ Tconv	9	11.0	165.5	0.314	8	57.8	98.5	0.093
CD69	CD69+ CD8+ T cells	9	68.0	134.3	0.374	8	84.0	140.0	0.263
CD69	CD69+ NK cells	9	71.0	106.5	0.314	8	-63.5	190.8	0.069
CD69	CD69+ γδ T cells	3	-43.5	723.5	0.285	4	-359.5	947.5	0.273
PD1	PD1+ CD4+ Tconv	9	16.5	36.0	0.086	8	-13.5	40.0	0.093
PD1	PD1+ CD8+ T cells	9	24.6	74.6	0.678	8	3.0	44.1	0.889
PD1	PD1+ NK cells	9	2.2	35.5	0.441	8	15.2	29.6	0.093
CD11b	Eosinophils	9	523.0	745.0	**0.008**	8	15.1	957.6	0.889
HLADR	Eosinophils	9	163.0	3008.0	0.866	7	77.0	2339.0	0.735
CD63	Eosinophils	9	898.0	1010.0	**0.008**	8	673.0	742.5	0.093
CD11b	Neutrophils	9	1602.0	476.0	**0.008**	8	1227.5	1656.0	**0.017**
HLADR	Neutrophils	9	-81.0	259.0	0.933	7	16.0	306.0	0.866
CD63	Neutrophils	9	419.0	191.0	**0.008**	8	307.0	713.5	**0.012**
CD11b	Monocytes	9	-1036.7	1525.0	0.086	8	-1102.8	1276.8	**0.025**
HLADR	Monocytes	9	-8722.0	24830.0	0.237	7	-9936.0	17353.0	**0.018**
CD63	Monocytes	9	-1800.0	4758.0	**0.038**	8	-2822.5	3136.5	**0.036**
CD127	CD56loCD16hi NK cells	9	65.0	147.0	0.086	8	25.0	83.5	0.327
CD127	CD56loCD16lo NK cells	9	-11.0	216.0	0.859	8	44.5	318.8	0.484
PD1	CD56loCD16hi NK cells	9	14.1	33.8	0.314	8	6.9	13.4	**0.017**
PD1	CD56loCD16lo NK cells	9	16.3	63.5	0.859	8	55.3	59.3	0.161
CD25	CD56loCD16hi NK cells	9	6.2	15.4	0.214	8	-0.9	4.5	0.401
CD25	CD56loCD16lo NK cells	9	8.5	29.6	0.594	8	-5.5	31.2	0.484

SE = stimulation effect (iSARS-iVERO)

Panel 1

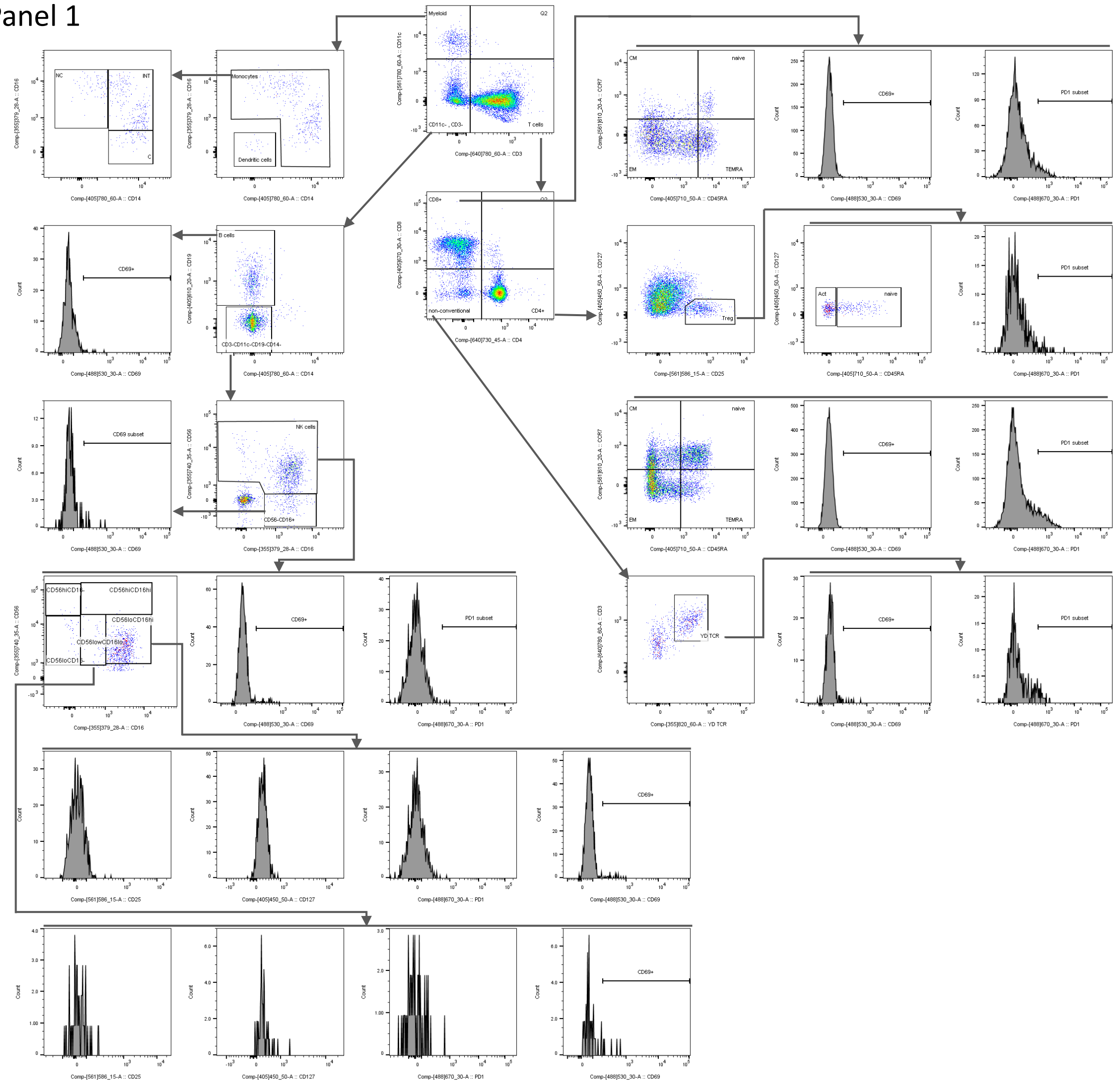

Panel 2

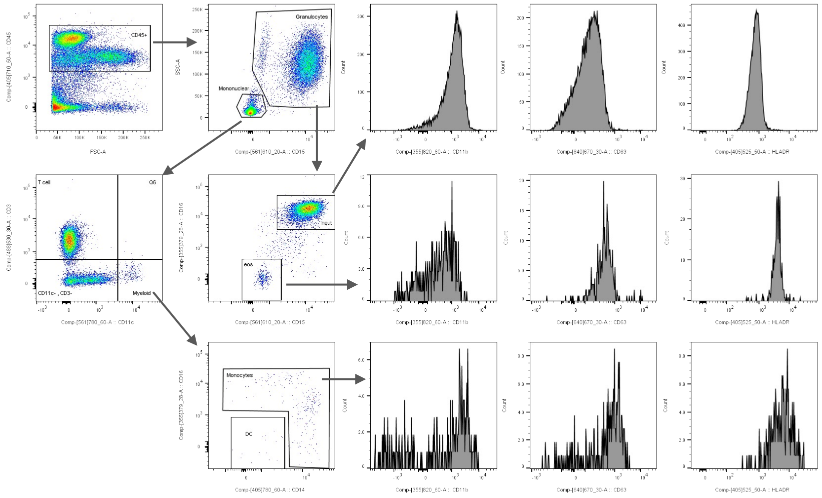

Supplementary figure 2: Single cell immunophenotyping manual gating strategies
